# Are Environmental Levels of Bisphenol A Associated with Reproductive Function in Fertile Men?

**DOI:** 10.1289/ehp.1002037

**Published:** 2010-05-21

**Authors:** Jaime Mendiola, Niels Jørgensen, Anna-Maria Andersson, Antonia M. Calafat, Xiaoyun Ye, J. Bruce Redmon, Erma Z. Drobnis, Christina Wang, Amy Sparks, Sally W. Thurston, Fan Liu, Shanna H. Swan

**Affiliations:** 1 Department of Obstetrics and Gynecology, School of Medicine and Dentistry, University of Rochester, Rochester, New York, USA; 2 University Department of Growth and Reproduction, University of Copenhagen, Rigshospitalet, Copenhagen, Denmark; 3 Division of Laboratory Sciences, National Center for Environmental Health, Centers for Disease Control and Prevention, Atlanta, Georgia, USA; 4 Department of Medicine and; 5 Department of Urologic Surgery, University of Minnesota Medical School, Minneapolis, Minnesota, USA; 6 Department of Obstetrics, Gynecology and Women’s Health, School of Medicine, University of Missouri, Columbia, Missouri, USA; 7 Division of Endocrinology, Department of Medicine, Harbor-UCLA Medical Center and Los Angeles Biomedical Research Institute, Torrance, California, USA; 8 Department of Obstetrics and Gynecology, University of Iowa, Iowa City, Iowa, USA; 9 University of Rochester School of Medicine and Dentistry, Department of Biostatistics and Computational Biology, Rochester, New York, USA

**Keywords:** bisphenol A, endocrine disruptors, male hormones, semen quality, xenoestrogens

## Abstract

**Background:**

Rodent and *in vitro* studies have demonstrated the estrogenicity of bisphenol A (BPA). However, few studies have examined the relationship between human exposure to BPA and male reproductive function.

**Objectives:**

We investigated the relationships between environmental BPA exposure and reproductive parameters, including semen quality and male reproductive hormones, in prospectively recruited fertile men.

**Methods:**

Participants (*n =* 375) were partners of pregnant women who participated in the Study for Future Families in four U.S. cities, and all of the men provided blood, semen, and urine samples. BPA was measured in urine. Serum samples were analyzed for reproductive hormones, including follicle-stimulating hormone, luteinizing hormone (LH), testosterone, inhibin B, estradiol, and sex hormone–binding globulin (SHBG), as well as the free androgen index (FAI). Semen analyses were performed according to World Health Organization criteria. Pearson correlations were used for unadjusted analyses, and multiple linear regression analyses were used to examine associations controlling for age, body mass index, smoking, ethnicity, urinary creatinine concentration, time of sample collection, and duration of abstinence.

**Results:**

After multivariate adjustment, we observed no significant associations between any semen parameter and urinary BPA concentration. However, a significant inverse association was found between urinary BPA concentration and FAI levels and the FAI/LH ratio, as well as a significant positive association between BPA and SHBG.

**Conclusions:**

Our results suggest that, in fertile men, exposure to low environmental levels of BPA may be associated with a modest reduction in markers of free testosterone, but any effects on reproductive function are likely to be small, and of uncertain clinical significance.

Bisphenol A (BPA) is used extensively in industry and commerce to manufacture polycarbonate plastics (which can be used in baby bottles, water storage tanks, or supply pipes) and components of food packaging (for example, in the lining of food cans), among other applications (Vandenberg et al. 2009). BPA can leach from some of these polymers into water or food products. In a representative subset of participants of the 2003–2004 National Health and Nutrition Examination Survey (NHANES), 92.6% of urinary samples presented detectable concentrations of BPA, indicating that exposure to BPA is common among the general population in the United States (Calafat et al. 2008).

BPA has been reported to have both estrogenic and antiandrogenic effects (Akingbemi et al. 2004; Lee et al. 2003; Wetherill et al. 2007). Several toxicological studies (Richter et al. 2007) have pointed out that rodents exposed to BPA during the prenatal or perinatal period show a large variety of adverse reproductive outcomes, including decreased epididymal weight and daily sperm production (Salian et al. 2009a, 2009b; vom Saal et al. 1998) and increased prostate weight (Nagel et al. 1997). Regarding prepubertal or pubertal exposures, rodent studies have described a dramatic decrease in testosterone (T) levels (Herath et al. 2004; Takao et al. 1999) and epididymal sperm counts (Herath et al. 2004) after BPA exposure. Adult male mice showed a significant reduction in testicular sperm counts, as well as epididymal sperm counts, after exposure to 25 or 100 ng/kg BPA (Al-Hiyasat et al. 2002). Tohei et al. (2001) reported that plasma concentrations of T were decreased and plasma concentrations of luteinizing hormone (LH) were increased in BPA-treated male adult rats compared with control rats.

Studies have reported secular declining trends in T levels in humans (Andersson et al. 2007; Travison et al. 2007) consistent with decreases in sperm concentration (Carlsen et al. 1992; Swan et al. 1997). It has been hypothesized that these changes might be the result, at least in part, of increasing human exposure to endocrine-disrupting compounds (EDCs) (Sharpe and Skakkebaek 2008). To our knowledge, only four studies have examined the relationship between reproductive function in men and BPA exposure. Takeuchi and Tsutsumi (2002) investigated the relationship between BPA exposure and hormone levels in men (*n* = 11) and women (*n* = 30), showing statistically significant positive correlations between serum BPA concentrations and total and free testosterone (FT) levels in both sexes. Hanaoka et al. (2002) studied men occupationally exposed to BPA during the application of epoxy resins (*n* = 42) compared with matched control workers without exposure (*n* = 42). Urinary concentrations of BPA were significantly higher in exposed workers, and the authors found a significant inverse association between urinary BPA concentrations and follicle-stimulating hormone (FSH) levels after controlling for age and alcohol use. Li et al. (2010) reported that BPA-exposed workers (*n =* 164) had higher risk of male sexual dysfunction, including, for example, reduced sexual desire or erectile difficulty compared with unexposed workers (*n =* 386). Recently, Meeker et al. (2010), examining a male population attending a fertility clinic (*n =* 167), reported inverse associations between urinary BPA concentrations and serum inhibin B levels and the estradiol (E_2_)/T ratio, and positive associations with serum FSH levels and the FSH/inhibin B ratio.

This is the first study to examine BPA and serum hormones in fertile men. It complements the earlier studies in occupational cohorts (with their higher exposure levels), and infertility clinic patients (enriched with men with altered hormone levels). Together with those previous studies, it is now possible to examine BPA across a range of exposures and reproductive potential.

The aim of the present study is to examine the associations between urinary BPA concentrations and reproductive function (semen quality and hormone levels) in a population of fertile men.

## Materials and Methods

### Study population

All men were participants in the Study for Future Families (SFF), a multicenter study of pregnant women and their partners conducted at prenatal clinics affiliated with university hospitals in four U.S. cities (Harbor-UCLA and Cedars-Sinai Medical Center in Los Angeles, CA; University of Minnesota Medical Center in Minneapolis, MN; University Physicians in Columbia, MO; and University of Iowa, Iowa City, IA) between 1999 and 2005. Methods for clinical examination, data collection, and semen analysis, which were standardized across centers, have been described previously (Swan et al. 2003). Couples were recruited at the prenatal clinic, and only those whose pregnancy was conceived without medical assistance were eligible. The men completed a questionnaire, received a physical examination, and gave blood, semen, and urine samples on the same day during the prenatal visit. Questions included demographics, recent fever, and history of sexually transmitted diseases, as well as lifestyle factors (smoking, and alcohol and caffeine consumption) and diet. Although 950 men participated in SFF, urine was not collected until the second year of the study, and approximately 85% of men provided a semen sample. This analysis is based on the 360 (39%) men for whom data on serum hormones and urinary BPA concentrations were available (*n =* 302 for complete covariates) and the 375 men with data on semen parameters and urinary BPA concentrations (*n =* 317 for complete covariates). The involvement of the Centers for Disease Control and Prevention (CDC) laboratory was limited and determined not to constitute engagement in human subjects research. This study has complied with all applicable requirements of the U.S. regulations. Human subject approvals were obtained from institutional review boards at all participating institutions. Participation of human subjects did not occur until after written informed consent was obtained.

### Urinary BPA measurements

The urinary concentrations of BPA in 375 men were determined at the Division of Laboratory Sciences, National Center for Environmental Health, CDC, which had no access to participant data. Urine samples were dispensed into smaller aliquots and frozen within 1 hr of collection, stored at −20°C, then shipped on dry ice overnight to the CDC, where they remained stored at or below −40°C until analysis. The total urinary concentration of BPA (free plus conjugated species) was measured using online solid-phase extraction (SPE) coupled to isotope dilution–high-performance liquid chromatography (HPLC)-tandem mass spectrometry (MS/MS) on a system constructed from several HPLC Agilent 1100 modules (Agilent Technologies, Wilmington, DE, USA) coupled to a triple quadrupole API 4000 mass spectrometer (Applied Biosystems, Foster City, CA, USA), as described in detail previously (Ye et al. 2005). Briefly, 100 μL urine was treated with β-glucuronidase/sulfatase (*Helix pomatia*, H1; Sigma Chemical Co., St. Louis, MO, USA) to hydrolyze the BPA-conjugated species. BPA was then retained and concentrated on a C18 reversed-phase size-exclusion SPE column (Merck KGaA, Darmstadt, Germany), separated from other urine matrix components using a pair of monolithic HPLC columns (Merck KGaA), and detected by negative ion-atmospheric pressure chemical ionization-MS/MS. The limit of detection (LOD) for BPA in a 0.1-mL urine sample was 0.4 μg/L. Low-concentration (~ 4 μg/L) and high-concentration (~ 20 μg/L) quality control materials, prepared with pooled human urine, were analyzed with standard, reagent blank, and unknown samples (Ye et al. 2005).

### Serum hormone analysis

Venous blood samples were drawn, and the serum was separated and frozen at −80°C. All samples were analyzed for hormones in the same laboratory at the Rigshospitalet (Copenhagen, Denmark). Samples were shipped to Copenhagen on dry ice and stored at −20°C until hormone analysis was performed. The methods used have been described previously (Asklund et al. 2007; Bang et al. 2005). Briefly, hormone assessments were done simultaneously to reduce intralaboratory variations. Serum levels of FSH, LH, and sex hormone–binding globulin (SHBG) were determined using time-resolved immunofluorometric assays (DELFIA; PerkinElmer, Skovlund, Denmark). Intra- and interassay variations were both < 5% in each of the three assays. T levels were determined using a time-resolved fluoroimmunoassay (DELFIA; PerkinElmer) with intra- and interassay variation < 8%. E_2_ was measured by radioimmunoassay (Pantex, Santa Monica, CA, USA) with an intraassay variation of < 8% and an interassay variation of < 13%. Inhibin B levels were determined by a specific two-sided enzyme immunometric assay (Oxford Bio-Innovation Ltd, Bicester, UK) with intra- and interassay variation of 13% and 18%, respectively. The free androgen index (FAI) was calculated as total T × 100/SHBG. The FT concentration was calculated using the equation of Vermeulen et al. (1999).

### Semen collection and analysis

Men collected semen samples by masturbation at the clinic, and almost all samples were processed within 45 min of collection. These methods are described in detail elsewhere (Brazil et al. 2004). Briefly, ejaculate volumes were estimated by specimen weight, assuming a semen density of 1.0 g/mL. Sperm concentration was evaluated by hemacytometer (Improved Neubauer; Hauser Scientific Inc., Horsham, PA, USA). In this analysis, the percent motile sperm refers to the percentage of sperm with any flagellar movement, whether twitching or progressive. Motility was also analyzed using the World Health Organization (WHO) 1999a, b, c, and d method, with forward motile sperm classified as a + b (WHO 1999). Seminal smears were prepared at the clinical centers and shipped to the Andrology Coordinating Center at the University of California, Davis, California, for Papanicalou staining, analysis, and storage. A single technician assessed sperm morphology using the strict morphology method, as recommended by the WHO (1999). In addition to the primary measures of semen quality (sperm concentration, volume, percent morphologically normal sperm, and percent motile sperm), we analyzed the total sperm count (volume × sperm concentration) and total motile count (volume × sperm concentration × motility). Men were requested to observe a 2- to 5-day abstinence period, and the importance of accurately reporting the actual abstinence period was stressed.

### Statistical analyses

The frequency distribution of serum hormones (except E_2_) and urinary BPA concentrations showed skewed (non-normal) distributions and were transformed using the natural log (ln) before analysis. We used Pearson correlation coefficients (*R*) and parametric tests to explore the relationship between each hormone level and urinary BPA concentration, both unadjusted and adjusted by urinary dilution (BPA concentration divided by creatinine concentration and expressed as micrograms per gram creatinine). Multiple linear regression analysis was then performed controlling for covariates previously noted to be associated with male reproductive function. The following covariates were included in the final model for hormone analysis: age, age squared, body mass index (BMI), smoking status (current smoker vs. not current smoker), ethnicity [(African American vs. others) because of relevance of racial and ethnic differences in sex steroids (Wang et al. 2007)], study center (Missouri vs. Iowa, Minnesota, or California; taking Missouri as a reference value because of lower semen quality compared with other centers), time of sample collection, and urinary creatinine concentration (ln-transformed). We adjusted for creatinine to account for urine dilution, because urine dilution can influence urinary BPA concentrations. For analyses of semen parameters, stressful life events (< 2 vs. ≥ 2 events) (Gollenberg et al. 2010), ejaculation abstinence time (hours), time from semen collection to initial processing (minutes), and time required to conduct semen analysis (minutes) were included in multivariate models. Ejaculation abstinence time is related to seminal volume and sperm concentration but not with sperm motility or morphology. Time from semen collection to initial processing and time required to conduct semen analysis are related to sperm motility but have no relationship with other sperm variables. Age, BMI, urinary creatinine concentration (ln-transformed), time of sample collection, ejaculation abstinence time, time from semen collection to initial processing, and time required to conduct semen analysis were modeled as continuous independent variable and all others as dichotomous variables. Most BPA concentrations were > LOD (89.7%); those < LOD were assigned a value of LOD divided by the square root of 2 (Hornung and Reed 1990). The level of statistical significance was set at 0.05. Data analysis was performed independently by two analysts, one using SAS version 9.1 (SAS Institute Inc., Cary, NC, USA) and one using SPSS version 17.0 (SPSS Inc., Chicago, IL, USA).

## Results

Mean age (± SD) and BMI (kilograms per meter squared; ± SD) of the included men were 31.9 ± 6.3 years (range, 18–53 years) and 28.3 ± 5.6 (range, 18.3–49.9), respectively. Forty-one percent of these men were overweight (BMI of 25–30), and an additional 29% were obese (BMI ≥ 30). Twenty-three percent of the men were smokers, 70% were white and 10% were African American. Table 1 shows the characteristics of semen parameters for our study population. Summary statistics for men’s reproductive hormones and urinary BPA concentrations (before creatinine adjustment) are shown in Table 2. Almost 90% (*n =* 235) of the urinary samples had concentrations of BPA > LOD (0.4 μg/L). The mean (± SD) urinary creatinine concentration was 144 ± 79.8 mg/dL (median*,* 138 mg/dL).

In unadjusted correlations, age was inversely correlated with T, FAI, and FT (*R* = −0.12, −0.32, −0.25, respectively; *p* < 0.05 for all) and positively correlated with FSH and SHBG (*R* = 0.13 and 0.15, respectively; *p* < 0.05 for both). BMI was negatively correlated with T, inhibin B, SHBG, and FT (*R* = −0.44, −0.35, −0.48, −0.23, respectively; *p* < 0.01 for all) and positively correlated with E_2_ (*R* = 0.24, *p* < 0.01). We also saw a smaller (positive) correlation between BMI and FAI, but it was driven by a small number of individuals with high BMI and very low SHBG. Other than these individuals, FAI and FT were highly correlated (data available on request). Current smokers had higher mean E_2_ levels than nonsmokers (93.5 pg/mL vs. 82.4 pg/mL, *p* ≤ 0.01).

Table 3 shows correlation coefficients for men’s reproductive hormones and urinary BPA concentrations (both unadjusted for creatinine and creatinine adjusted). These univariate associations differed somewhat before and after creatinine adjustment. We observed significant inverse correlation between creatinine-adjusted urinary BPA concentrations and FAI level, FAI/LH ratio, and FT/LH ratio. All of these measures are related to available T or SHBG levels, but neither T nor SHBG were correlated with urinary BPA. We also found a suggestive inverse correlation between creatinine-adjusted BPA concentrations and FT (*p* = 0.08) and T/LH ratio (*p* = 0.054). Correlation coefficients between semen parameters and unadjusted and creatinine-adjusted BPA concentrations are shown in Table 4. Seminal volume was negatively correlated with both unadjusted and creatinine-adjusted urinary BPA concentrations, but no other semen parameters were appreciably associated with BPA concentration.

Table 5 shows the results of the multivariate analysis for men’s reproductive hormone and urinary BPA concentrations. Consistent with the unadjusted results, we saw no significant associations between BPA concentration and any of the six hormones. After adjustment for possible confounders, we observed significant inverse associations between urinary BPA and both FAI and the FAI/LH ratio [regression coefficient (β) = −0.05; 95% confidence interval (CI), −0.09 to −0.004; and β= −0.11; 95% CI, −0.18 to −0.03, respectively], although not for FT, a measure of free T calculated from concentrations of total T, albumin, and SHBG along with T-binding constants to albumin and SHBG. There was a significant positive association between serum SHBG levels and urinary BPA concentration (β = 0.07; 95% CI, 0.007–0.13). We also observed suggestive inverse associations between urinary BPA concentration and both FT/LH ratio and seminal volume (Table 6), and a suggestive positive association between BPA concentrations and serum LH levels. We reran our statistical models excluding two outliers (subjects with extremely high concentrations of urinary BPA) and observed somewhat weaker but still statistically significant associations (data not shown). An interquartile increase in urinary BPA concentrations (2.1 μg/L) for a 32-year-old nonsmoker with BMI of 28 kg/m^2^ would be predicted to decrease FAI levels and FAI/LH ratio around 6% and 11%, respectively. After adjustment, there were no significant associations between urinary BPA concentrations and any of the semen parameters examined (Table 6).

## Discussion

To our knowledge, this is the first study to examine the relationships between urinary BPA concentrations and markers of reproductive function (semen quality and hormone levels) in fertile men. We saw a significant association with FAI/LH (but not T/LH or FT/LH) and FAI after controlling for covariates.

Our results are divergent from those reported previously by Takeuchi and Tsutsumi (2002) and Hanaoka et al. (2002). Takeuchi and Tsutsumi (2002) used an enzyme-linked immunosorbent assay (ELISA) to measure BPA in the serum of men and women, showing statistically significant positive correlations between BPA concentrations and total T and FT levels in all subjects. However, these differences may be related to differences both in population size and the different methodological approaches to measuring BPA (i.e., ELISA versus online SPE-HPLC-MS/MS; human serum versus urine). ELISA is known to be a very sensitive method but is not as specific as MS/MS. It is possible that with ELISA, substances other than BPA and its conjugates—including other bisphenols—can be detected (Vandenberg et al. 2010). Hanaoka et al. (2002) examined men occupationally exposed to BPA and reported that urinary concentrations of BPA, measured by HPLC with electrochemical detection, were significantly higher in exposed workers than in controls; the authors also found a significant inverse association between urinary BPA concentrations and serum FSH levels. Nevertheless, FT levels (measured by radio immunosolvent assay) did not differ between the two groups.

In the present study we did not observe any relationship between serum FSH levels and urinary BPA concentrations, but we did observe an inverse association between FAI and urinary BPA concentrations. Hanaoka et al. (2002) speculated that BPA binds to estrogen receptor (ER) in the pituitary gland, resulting in direct suppression of FSH secretion; this is based on studies that have found ERs in the pituitary gland (Pelletier 2000) and that E_2_ directly inhibits gonadotropin secretion at the pituitary level in men (Finkelstein et al. 1991). Recently, Meeker et al. (2010) evaluated men referred for fertility work-up; in a subset who collected at least two urine samples (*n* = 75), the authors observed inverse associations between urinary BPA and FAI, E_2_, and thyroid-stimulating hormone levels. However, when they analyzed the entire cohort of men (*n =* 167), all of whom collected a single urine sample, urinary BPA concentrations were inversely associated only with serum inhibin B levels and positively associated with serum FSH levels.

There is a large and consistent body of literature showing BPA to be estrogenic (Richter et al. 2007; Wetherill et al. 2007), and it is considered by some researchers to be one of the most potent reproductive toxicants among EDCs (Maffini et al. 2006). Animal and *in vitro* studies have shown that BPA is associated with the induction of testicular toxicity in neonatal, pubertal, and adult rodents (Richter et al. 2007; Wetherill et al. 2007). Akingbemi et al. (2004) described an inhibitory effect of BPA on testicular steroidogenesis at low exposure levels in pubertal rats, which they ascribed to an ER-mediated effect. In addition to its antiandrogenic effects through ER-mediated down-regulation of steroidogenesis (and thereby T production), BPA might also act as an androgen receptor antagonist, preventing endogenous androgens from regulating androgen-dependent transcription (Wetherill et al. 2007). The disruption of the androgen receptor–androgen interaction has been speculated to be significant in eliciting adverse effects on the male reproductive system, including sexual dysfunctions (Li et al. 2010).

Our results suggest that FAI levels—one of the markers of the biologically active T—may be somewhat decreased by BPA exposure. Nonetheless, the magnitude of the effect of BPA in the present cross-sectional study on FAI was small compared with, for example, the diurnal variation in this parameter in healthy young men (Bremner et al. 1983; Diver et al. 2003). However, the effects of long-term exposure to BPA in adult men are unknown, and we cannot rule out that such exposure may exert an effect on their hormone production. It is also important to point out that we are certainly exposed to a mixture of EDCs, and the cumulative effects that these chemicals may exert in combination are only now being studied (Kortenkamp 2008).

In the present study, we observed an inverse association between BPA exposure and FAI levels *in vivo* with no apparent compensatory increase in serum gonadotropins, as shown by the lack of significant associations with LH and FSH. However, we cannot rule out a possible compensatory increase in serum gonadotropins in a larger study or another kind of study population. The fact that FAI and SHBG are statistically associated with urinary BPA concentrations while T individually is not suggests that the small associations observed between these hormones and BPA could have resulted from an increase in SHBG. The regulation of SHBG is not completely understood, but androgen action lowers serum SHBG, whereas estrogen action increases it. The increase in SHBG levels we report here could be a direct result of the estrogenic action of BPA. Alternatively, it is possible that BPA acts by decreasing androgen action through ER-mediated decrease in steroid production. However, there might be noncausal explanations for the association with SHBG as well.

We speculate that BPA *in vivo* may act at several levels. In addition to the alteration of markers of androgenic action, the endocrine feedback loop (mainly the hypothalamic– pituitary–target organ axis) could also be affected by BPA, showing no compensatory mechanisms of increased LH or FSH. Alternatively, because there is no change in T with BPA (adjusted β = 0.01; 95% CI, −0.04 to 0.06), this suggests that the signal is insufficient to trigger the feedback mechanism to the hypothalamus and pituitary.

We observed no significant associations between semen parameters and BPA exposures in our population of fertile men. It is important to remember that spermatogenesis takes 70–80 days, and a clear association between semen parameters and relatively low and chronic urinary BPA concentrations may be difficult to assess. It is also possible that in men with compromised semen quality (such as those attending an infertility clinic) or men exposed occupationally to higher concentrations of BPA, some associations may be seen that are absent in our fertile population.

Urinary concentrations of BPA in our subjects were only about half as high as those reported in a national sample of U.S. men (CDC 2008). For example, the 25th, median, and 75th percentile values for BPA in adult males (range, 18–65 years of age) from NHANES 2003–2004 were 1.5, 3.1, and 6.1 μg/L, respectively, compared with 0.80, 1.7, and 3.0 μg/L in the present study. Whether the lower BPA urinary concentrations in the men in our study compared with men in NHANES was related to their fertility is not a question we could examine in this data set. Recently, in an analysis of NHANES data, Stahlhut et al. (2009) showed that BPA was detectable in urine up to 24 hr after the last meal, suggesting substantial nonfood exposure, accumulation in body tissues such as fat, or both. In addition, we cannot rule out some level of occupational exposure to BPA in our study population, but it is not likely because of the relatively low levels mentioned above.

Our study was limited because we selected only fertile men; therefore, our results should be applied only to that type of population. Also, we used a single urine sample to assess BPA exposure and a single serum sample to describe hormone function. However, Mahalingaiah et al. (2008) maintained that, despite within-person variability in urinary BPA concentrations, a single sample is predictive of long-term exposure (over weeks to months) and provides good sensitivity to classify individuals in epidemiologic studies. Exposure measurement error is likely in the present study; if this measurement error was nondifferential, we could expect that, on average, the effect estimates might be biased toward the null. Similarly, a single sample can be used to classify men’s reproductive hormones (Bjornerem et al. 2006). As with all studies of semen quality, small participation rates, potential selection bias, and uncontrolled confounding are of concern. In a previous study (Swan et al. 2003), we examined selection bias by comparing questionnaire data on time to pregnancy and history of infertility, as well as demographics, of study subjects and nonparticipants and of men who did and did not give semen samples. Reassuringly, we found little evidence that those populations differed.

## Conclusions

Our results suggest that low environmental levels of BPA in a large group of prospectively recruited fertile men is not associated with sperm quality but is weakly associated with markers of androgenic action. These results may reflect the estrogenic effect of BPA, which we hypothesize could be related to an inhibitory effect on testicular steroidogenesis. However, associations between BPA exposure and measures of reproductive function in fertile men were small and of uncertain clinical significance. Consequently, further studies will be needed to elucidate the associations between BPA exposure and reproductive function in men.

## Figures and Tables

**Table 1:** Characteristics of semen parameters for our study population (*n* = 375).

Variable	Mean ± SD (median)
Seminal volume (mL)	3.9 ± 1.7 (3.6)
Sperm concentration (× 10^6^/mL)	79.9 ± 58.9 (67.1)
Motile sperm (%)	51.3 ± 11.9 (53.0)
Morphologically normal sperm (%)	10.7 ± 5.2 (10.0)
Total motile count (× 10^6^)	139 ± 112 (117)
Total sperm count (× 10^6^)	295 ± 220 (242)

**Table 2:** Summary statistics for the serum concentrations of men’s reproductive hormones (*n* = 360) and urinary concentrations of BPA (*n* = 375).

Variable	Geometric mean	Percentile				
		5th	25th	50th	75th	95th
FSH (IU/L)	2.9	1.2	2.0	2.9	4.1	6.5
LH (IU/L)	3.3	1.7	2.6	3.3	4.4	6.2
T (nmol/L)	17.8	9.3	14.0	18.1	23.3	31.7
Inhibin B (pg/mL)	207	105	161	217	269	369
E_2_ (pmol/L)	79.5	41.0	66.0	83.0	103	132
SHBG (nmol/L)	27.3	12.0	20.3	28.0	37.0	58.0
FAI	65.1	36.1	50.7	66.1	81.8	118
FT	11.6	6.2	9.5	11.8	14.6	19.1
FAI/LH	19.7	8.2	14.1	19.2	28.8	47.0
FT/LH	3.5	1.6	2.5	3.7	4.9	7.1
T/E_2_ ratio	0.22	0.10	0.17	0.23	0.29	0.50
T/LH ratio	5.4	2.5	3.8	5.5	7.5	10.9
BPA (μg/L)^a^	1.5	< LOD	0.80	1.7	3.0	6.5

a LOD for BPA was 0.4 μg/L; 89.7% of the urinary concentrations were > LOD.

**Table 3:** Correlation coefficients for men’s reproductive hormones and urinary BPA concentrations (univariate analysis) (*n* = 360).^a^

	BPA concentration (μg/L)		Creatinine-adjusted BPA concentration (μg/g)	
Variable	*R* (95% CI)	*p*-Value	*R* (95% CI)	*p*-Value
FSH (IU/L)	−0.004 (−0.11 to 0.10)	0.95	0.06 (−0.04 to 0.16)	0.28
LH (IU/L)	0.09 (−0.01 to 0.19)	0.10	0.08 (−0.02 to 0.18)	0.14
T (nmol/L)	−0.05 (−0.15 to 0.05)	0.39	−0.05 (−0.15 to 0.05)	0.37
Inhibin B (pg/mL)	−0.11 (−0.21 to −0.01)	0.04	−0.06 (−0.04 to 0.16)	0.26
E_2_ (pmol/L)	0.07 (−0.03 to 0.17)	0.22	0.03 (−0.07 to 0.13)	0.60
SHBG (nmol/L)	−0.02 (−0.12 to 0.08)	0.72	0.05 (−0.05 to 0.15)	0.37
FAI	−0.02 (−0.12 to 0.08)	0.67	−0.11 (−0.21 to −0.01)	0.04
FT	−0.05 (−0.15 to 0.05)	0.38	−0.09 (−0.19 to 0.01)	0.08
FAI/LH	−0.08 (−0.18 to 0.02)	0.14	−0.13 (−0.23 to −0.03)	0.01
FT/LH	−0.11 (−0.21 to −0.01)	0.04	−0.13 (−0.23 to −0.03)	0.01
T/E_2_ ratio	−0.09 (−0.19 to 0.01)	0.11	−0.05 (−0.15 to 0.05)	0.36
T/LH ratio	−0.11 (−0.21 to −0.01)	0.04	−0.10 (−0.20 to 0.01)	0.05

a BPA and all hormones except for E_2_ were ln-transformed.

**Table 4:** Correlation coefficients between semen parameters and urinary BPA concentrations (univariate analysis) (*n* = 375).^a^

	BPA concentration (μg/L)		Creatinine-adjusted BPA concentration (μg/g)	
Variable	*R* (95% CI)	*p*-Value	*R* (95% CI)	*p*-Value
Seminal volume	−0.16 (−0.26 to −0.06)	< 0.01	−0.11 (−0.21 to −0.01)	0.03
Sperm concentration^b^	−0.03 (−0.13 to 0.07)	0.57	−0.003 (−0.10 to 0.10)	0.95
Motile sperm (%)	−0.004 (−0.11 to 0.10)	0.94	−0.08 (−0.18 to 0.02)	0.11
Morphologically normal sperm (%)^b^	0.05 (−0.05 to 0.15)	0.33	0.02 (−0.08 to 0.12)	0.72
Total motile count	−0.09 (−0.19 to 0.01)	0.07	−0.08 (−0.18 to 0.02)	0.12
Total sperm count^b^	−0.11 (−0.21 to −0.01)	0.03	−0.07 (−0.17 to 0.03)	0.19

a BPA, sperm concentration, and total motile count were ln-transformed.

b *n* = 374.

**Table 5:** Multivariate analysis for men’s reproductive hormones and urinary BPA concentrations (*n* = 302).^a^

	Urinary BPA concentrations
Variable	β (95% CI)
FSH	0.04 (−0.04 to 0.12)
LH	0.05 (−0.01 to 0.12)
T	0.01 (−0.04 to 0.06)
Inhibin B	−0.01 (−0.07 to 0.04)
E_2_	0.49 (−3.1 to 4.1)
SHBG	0.07 (0.007 to 0.13)*
FAI	−0.05 (−0.09 to −0.004)*
FT	−0.01 (−0.06 to 0.03)
FAI/LH	−0.11 (−0.18 to −0.03)**
FT/LH	−0.07 (−0.13 to 0.001)
T/E_2_ ratio	0.01 (−0.05 to 0.08)
T/LH ratio	−0.04 (−0.11 to 0.03)

BPA, urinary creatinine concentration, and all hormones except E_2_ were ln-transformed.

a Controlling for age, age squared, BMI, smoking status (current smoker vs. not current smoker), ethnicity (African American vs. others), study center (Missouri vs. Iowa, Minnesota, or California), urinary creatinine concentration, and time of sample collection.

* *p* ≤ 0.05.

** *p* ≤ 0.01.

**Table 6:** Multivariate analysis for semen parameters in men and urinary BPA concentrations (*n* = 317).

	Urinary BPA concentration
Variable	β (95% CI)
Seminal volume^a^	−0.18 (−0.40 to 0.01)
Sperm concentration^a^	0.01 (−0.08 to 0.10)
Motile sperm (%)^b^	−0.38 (−1.66 to 0.90)
Morphologically normal sperm (%)^c^	0.16 (−0.45 to 0.77)
Total motile count^d^	−0.05 (−0.17 to 0.70)
Total sperm count^a^	−0.04 (−0.14 to 0.06)

*n* = 315 for seminal volume, and *n* = 314 for sperm concentration and total sperm and total motile count. BPA, sperm concentration, and total motile count were ln-transformed.

a Controlling for age, age squared, BMI, study center (Missouri vs. Iowa, Minnesota, or California), stressful life events (< 2 vs. ≥ 2 events), and ejaculation abstinence time.

b Controlling for age, age squared, BMI, study center (Missouri vs. Iowa, Minnesota, or California), stressful life events (< 2 vs. ≥ 2 events), time from semen collection to initial processing, and time required to conduct semen analysis.

c Controlling for age, age squared, BMI, study center (Missouri vs. Iowa, Minnesota, or California), and stressful life events (< 2 vs. ≥ 2 events).

d Controlling for age, age squared, BMI, study center (Missouri vs. Iowa, Minnesota, or California), stressful life events (< 2 vs. ≥ 2 events), ejaculation abstinence time, time from semen collection to initial processing, and time required to conduct semen analysis.
